# Post-Kala-azar Dermal Leishmaniasis in Nepal: A Retrospective Cohort Study (2000–2010)

**DOI:** 10.1371/journal.pntd.0001433

**Published:** 2011-12-20

**Authors:** Surendra Uranw, Bart Ostyn, Arpana Rijal, Saru Devkota, Basudha Khanal, Joris Menten, Marleen Boelaert, Suman Rijal

**Affiliations:** 1 Department of Internal Medicine, B.P. Koirala Institute of Health Sciences, Ghopa, Dharan, Nepal; 2 Department of Public Health, Institute of Tropical Medicine, Antwerp, Belgium; 3 Department of Dermatology, B.P. Koirala Institute of Health Sciences, Ghopa, Dharan, Nepal; 4 Department of Microbiology, B.P. Koirala Institute of Health Sciences, Ghopa, Dharan, Nepal; London School of Hygiene and Tropical Medicine, United Kingdom

## Abstract

**Introduction:**

Post-kala-azar dermal leishmaniasis (PKDL) is a cutaneous complication appearing after treatment of visceral leishmaniasis, and PKDL patients are considered infectious to sand flies and may therefore play a role in the transmission of VL. We estimated the risk and risk factors of PKDL in patients with past VL treatment in south-eastern Nepal.

**Methods:**

Between February and May 2010 we traced all patients who had received VL treatment during 2000–2009 in five high-endemic districts and screened them for PKDL-like skin lesions. Suspected cases were referred to a tertiary care hospital for confirmation by parasitology (slit skin smear (SSS)) and/or histopathology. We calculated the risk of PKDL using Kaplan-Meier survival curves and exact logistic regression for risk factors.

**Results:**

Out of 680 past-treated VL patients, 37(5.4%) presented active skin lesions suspect of PKDL during the survey. Thirty-three of them underwent dermatological assessment, and 16 (2.4%) were ascertained as probable (2) or confirmed (14) PKDL. Survival analysis showed a 1.4% risk of PKDL within 2 years of VL treatment. All 16 had been previously treated with sodium stibogluconate (SSG) for their VL. In 5, treatment had not been completed (≤21 injections). Skin lesions developed after a median time interval of 23 months [interquartile range (IQR) 16–40]. We found a higher PKDL rate (29.4%) in those inadequately treated compared to those who received a full SSG course (2.0%). In the logistic regression model, unsupervised treatment [odds ratio (OR) = 8.58, 95% CI 1.21–374.77], and inadequate SSG treatment for VL in the past (OR = 11.68, 95% CI 2.71–45.47) were significantly associated with PKDL.

**Conclusion:**

The occurrence of PKDL after VL treatment in Nepal is low compared to neighboring countries. Supervised and adequate treatment of VL seems essential to reduce the risk of PKDL development and active surveillance for PKDL is needed.

## Author Summary

Post-kala-azar dermal leishmaniasis (PKDL) is a skin disorder seen in patients treated for *Leishmania donovani* visceral leishmaniasis (VL), a neglected tropical disease that is fatal if left untreated. In the Indian subcontinent, PKDL is seen in 5–10% of all past VL cases and is also reported in some without history of VL. As persons with PKDL do not feel sick, the disease has only cosmetic significance for the individual and treatment is rarely sought. However, PKDL lesions harbour parasites and therefore could represent a source of transmission, through the bite of female sand flies. Our study shows that the occurrence of PKDL in patients with past treated VL is low in Nepal compared to neighboring countries. Treatment of the original VL episode with SSG (sodium stibogluconate), inadequate treatment and treatment on ambulatory basis were significantly associated with PKDL. Though SSG has since been replaced by other drugs, counseling and supervision of adherence to the prescribed VL treatment is of vital importance to reduce risk of treatment failure and relapse as well as later development of PKDL. Policy makers should include surveillance and case management of PKDL in the VL elimination program.

## Introduction

Post-kala-azar dermal leishmaniasis (PKDL) is a late complication of visceral leishmaniasis (VL), which usually appears several months after treatment of a VL episode. PKDL is seen in areas where *L.donovani* is endemic i.e. in Asia (India, Nepal and Bangladesh) and in east Africa (Ethiopia, Kenya and Sudan [1]. In the Indian subcontinent, *L.donovani* is transmitted by the bite of a female sand fly of the *Phlebotomus argentipes* species, and the transmission cycle is considered to be anthroponotic with humans as the only known infection reservoir [2]. In Nepal, the standard treatment for VL with SSG was 20 mg/kg/day for 30 days without any upper limit recommended by WHO and drug was provided by the program to all government hospital in the endemic area. Due to associated toxicity and emerging drug resistance, SSG has been replaced in 2007 by Miltefosine 50 mg BID.

PKDL is characterized by a spectrum of skin lesions ranging from hypo-pigmented macules, papules to nodules or combinations over the trunk and face that can be easily confused with other skin conditions such as vitiligo or leprosy [1], [3], [4]. So far, no convincing clinical predictors for PKDL have been identified [1] and its origin is believed to be multi-factorial and complex [5]. In Sudan, PKDL is more commonly reported in inadequately or irregularly treated VL cases [6]. PKDL is also sporadically reported in individuals without past history of VL [1], [7].

The incidence of PKDL varies from country to country for reasons that are not entirely clear. In Sudan, PKDL was described in 50–60% of cured VL patients within weeks to a few months after treatment [1]. In Bangladesh, a cross-sectional survey carried out in 2009 of patients who suffered from VL in 2002–2007 found 10% of them with active or past PKDL usually occurring within 36 months after VL treatment [8], [9]. In India, PKDL is reported in 5–10% of patients treated for VL usually after an interval of 2 to 4 years [3] and in 15–20% of PKDL cases there is no previous history of VL [3]. In Nepal VL is endemic in the south eastern Terai plains bordering the highly endemic districts of Bihar state of India, but systematic epidemiological data on PKDL are still lacking.

PKDL patients have probably epidemiological importance in VL transmission as the lesions can harbour a large amount of *Leishmania parasites*, and as such could constitute a reservoir in the community capable of triggering a new epidemic [9]. As PKDL causes little or no clinical discomfort, and PKDL treatment with intramuscular SSG injections is long (3–4 months), painful and cumbersome, few patients seek treatment [1], [5], [10]–[13].

Since 2005, the government of Nepal is involved in a collaborative effort with India and Bangladesh to reduce VL incidence to less than 1 per 10 000 population by 2015 [14]. PKDL is not addressed so far in this elimination initiative, which poses a threat to its success [15]. Better information on the epidemiology and burden of PKDL might help the national policy makers and health authorities to develop regional or national guidelines for its surveillance, control and treatment.

We therefore conducted a retrospective cohort study and studied the probability and risk factors for PKDL development in past treated VL cases in five districts of south-eastern Nepal.

## Materials and Methods

### Study area and population

The study was conducted from February to May 2010, in the districts of Jhapa, Morang, Sunsari, Saptari, and Siraha, known to be highly endemic for VL, with incidence rates of 2.0–4.0 per 10,000 person-years (pyr) in 2006/2007. All VL cases that were notified by these 5 districts during the period 2000–2009 were taken as the study population. Information to trace the past treated VL cases (pVL) at household level was obtained through the district public health office (DPHO) of the districts and the VL patient database (2000–2009) of B.P. Koirala Institute of Health Sciences (BPKIHS). BPKIHS is a university hospital located in Sunsari district that serves as the referral hospital for the region. The VL treatment centre maintains a patient register with clinical and epidemiological information. All pVL patients were approached at their residence by our field workers along with the vector control officer working at the DPHO. Written informed consent was obtained from pVL patients before including them into the study.

### Study design and sample size

The study was set up as a retrospective cohort study. Power calculations were as follows. We defined a priori 2 groups of patients according to their VL treatment experience: (i) VL patients treated in the government/private health facilities where most of the cases were treated on an ambulatory basis (unsupervised) by local medical assistants, and (ii) VL patients treated at BPKIHS where VL cases mostly were hospitalized for the full duration of treatment (supervised). To detect a risk ratio of 5, a sample of 332 was required in each group (treatment in government/private health facilities vs at BPKIHS) with the two-sided confidence level of 5%, a power of 80%, and a 1% expected frequency of PKDL in unexposed (i.e. supervised treatment).

### Data collection

#### Screening for PKDL

Before the initiation of the survey, all interviewers were trained on the origin of PKDL, the clinical picture of PKDL lesions, the data collecting tools and methods to screen for suspected PKDL cases.

Trained field workers identified the pVLs at their residence, asked for informed consent and interviewed them using a structured questionnaire. Detailed information on demographic characteristics and past VL treatment (*i.e.* date of onset of VL, health seeking behaviour, place of treatment, date of treatment, drug of choice, dosage and duration, clinical setting, hospitalization during treatment and adherence) was obtained. All pVL cases were asked about the current or past presence of hypo-pigmented skin lesions. This question was supported by a pictorial album showing colour pictures of real PKDL skin lesions. No blood or other samples were taken. All individuals with PKDL-like skin lesions found during the survey, they were asked about the time of onset of hypo- pigmented lesions, mode of onset and treatment seeking behaviour. The head of household and the pVL case were also asked whether other individuals within the family had suffered from VL in the past, and if any of them currently had skin rash. If during the survey, persons other than those listed in the “past VL cases list” presented spontaneously with suspected PKDL lesions, the field workers were asked to refer these cases for further diagnostic workup as well.

Individuals with current (or past) PKDL-like skin lesions were counselled and referred to BPKIHS for a complete diagnostic workup and management. A personalized visiting card with the name of the health staff member to contact upon their arrival was given so that their admission at the hospital would be facilitated. The list of suspected PKDL cases was used to check for completeness of attendance, and those who did not attend BPKIHS within 2 months were revisited in their homes a second time to facilitate the case confirmation.

PKDL cases were treated as per national guideline for kala-azar elimination. Treatment and treatment outcomes of PKDL will be documented in a separate study. Costs related to the patient management (travel, diagnostic workout, medicines, hospitalization and minimal care taker allowance for food) were provided, regardless of the outcome of the diagnosis.

### Confirmation procedure

A dermatologist examined all hypo-pigmented skin lesions cases referred to BPKIHS clinically and took samples for slit skin examination (SSE) and punch biopsy for histopathology for *L. donovani*. Differential diagnosis including leprosy, skin tuberculosis and other fungal infections was done in parallel. Every suspect was also tested by the rK39 immunochromatographic test for VL (see below). Based on this assessment, **a probable PKDL** case was defined as a person having a past history of VL and multiple hypo-pigmented skin lesions (macules, papules, plaques or nodules) with a positive rK39 test but negative for *L. donovani* in SSE or histopathological examination. **A confirmed PKDL** was defined as a patient with multiple hypo pigmented macules, papules, plaques or nodules, who was parasite positive in SSE or biopsy.

### Laboratory methods

#### Slit skin smear for *L.donovani* and *Mycobacterium leprae*

Slit skin smear test is routinely done in the department of dermatology for parasitological examination of PKDL and leprosy, as follows. The affected area of the skin was cleaned and dried. The edge of the lesion was squeezed firmly between the finger and the thumb to drain the blood from the area. Using a sterile scalpel blade no. 21, a 3–4 mm incision of 3 mm depth was made into the dermis. The slit skin smear was done by a trained medical officer under the close supervision of a dermatologist. One slide was stained with Giemsa and examined for *Leishmania donovani* (LD) bodies. The other was stained with Ziehl-Neelsen (Z-N) using 5% sulphuric acid, H_2_SO_4_) for *Mycobacterium leprae* and examined under oil immersion lens using 100× oil immersion objectives. Both slides were examined by two separate experts for confirmation.

#### Skin punch biopsy

Punch biopsy is routinely performed in the Department of Dermatology of BPKIHS for histopathological examination of PKDL. The skin lesions to be biopsied were selected, cleaned, dried and anesthetized to limit the discomfort. Biopsy was performed using a sterile circular blade no. 21 or trephine attached to a pencil-like handle. The instrument was rotated down through the epidermis and dermis and punch biopsy yields a cylindrical core of tissue. The biopsies were gently handled (usually with a needle) to prevent crush. The wound was closed, if necessary, with one or two interrupted nylon sutures. The diagnosis of PKDL was confirmed by standard histological examination (Giemsa stain) and quantification of *Leishmania* spp.

#### rK39

A trained medical officer performed the rK39 immunochromatographic test (Kala-azar Detect™ Rapid Test, *In*Bios International, Seattle, WA, USA) as per the manufacturer's instructions.

#### Statistical analysis

Data were cross-checked for data entry errors by performing cross-tabulation and verifying against the source document.

We calculated the risk of PKDL (probable+confirmed) among past treated VL cases over time using life table-methods and Kaplan-Meier survival curves. The log-rank test was used for comparison of survival data of different groups to correct for unequal follow-up times. We assessed risk factors for PKDL development using single and multiple exact logistic regression models. Exact logistic regression was used as it allows testing and estimation of risk factors with sparse data. A backward elimination strategy was used to identify factors independently associated with disease; probability for removal was set at 5%. Data were analysed using EPI info software package version 3.5.1 (Centre for Disease Control and Prevention, Atlanta, Georgia US) and Stata 10.1 (StataCorp LP, College Sation, Texas US).

### Ethical considerations

The Institutional Ethical Review Board of the BPKIHS Dharan, Nepal, and the corresponding bodies at the University Antwerp (UZA), Antwerp, Belgium reviewed and approved the study protocol. Informed consent forms were developed in the national language and informed consent was obtained from individuals and from parents for children and adolescents. Written approval also was obtained from the authority of District Public Health Offices (DPHO) when information about past VL was collected from respective districts.

## Results

### Characteristics of study population

From the records, a total of 742 past VL patients (pVLs) were identified clustered in 17 highly endemic villages. Field workers succeeded in tracing 680 (91.6%). All subjects consented to the study and were interviewed and screened for PKDL-like skin lesions at their residence [Table 1]. 560 (82.4%) had been treated with SSG (20 mg/kg/d for 30 consecutive days), 66 (9.7%) with Amphotericin B, and 54 (7.9%) with Miltefosine. In the SSG group, 17 (3%) had not completed the treatment (<21 injections). Half of the VL cases (347 or 51.0%) had been treated at BPKIHS hospital under supervision during the period of treatment and all had received complete treatment; 333 (49.0%) were treated at government/private hospitals on ambulatory basis, including 17 who had been treated in India. In our study, 370 (54.4%) were male, and the median age of the study population was 28; inter quartile range (IQR, 15–40) year and the large majority (75.4%) was aged ≥15 years.

**Table 1:** Risk of PKDL development in past treated VL patients, life table analysis over time.

Time intervals	No. of PKDL	Cumulative PKDL rate	95% CI
0–<2 years	9	0.014	0.007–0.027
2–<4 years	5	0.025	0.015–0.042
4–<8 years	2	0.036	0.021–0.062

### Screening of PKDL in past VL patients

Among the 680 pVL cases screened 37 (5.4%) individuals showed hypo pigmented skin lesions. . No pVL patient in the study reported any skin disorder that since had disappeared spontaneously. All the 37 individuals with current skin lesions were referred to BPKIHS, as well as 2 others with suspect skin lesions who presented themselves spontaneously to the surveyors: one without history of clinical VL and a second with VL prior to 2000.

**Figure 1:**
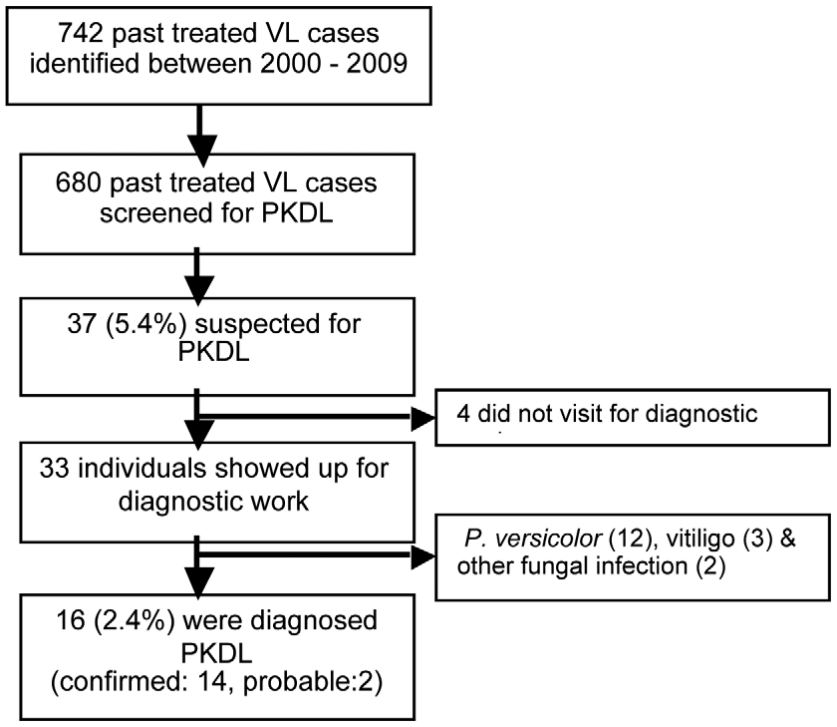
Recruitment and outcome of PKDL screening survey. Flow chart of study population: from number of patients screened to number of PKDL cases identified.

Thirty-three of the 37 referred pVLs reached the BPKIHS hospital and 16 (2.4% of 680) were diagnosed as PKDL (probable: 2 and confirmed: 14) [Figure 1]. The other 17 were diagnosed as *Pityriasis versicolor* (12), other fungal infection (2), and vitiligo (3). PKDL was also confirmed in the person without VL history, but not in the person who spontaneously reported with VL before 2000. All 37 suspects tested positive in the rK39 rapid diagnostic test. HIV status was not tested.

### Characteristics of pVL patients with probable and confirmed PKDL

The median age of the 16 PKDL patients was 23.5 years (IQR, 16–40), and majority (14/16) was aged ≥15 years. All PKDL cases had previously been treated for VL with SSG and no PKDL was found in the treatment with Amphotericin B and Miltefosine. Five had not completed treatment (less than 21 injections instead of the required 30). Most of the patients (15) had been treated at government/private hospitals on ambulatory basis. The overall prevalence of PKDL in SSG treatment was 2.9%, 0.3% in supervised and 4.5% in unsupervised treatment. The median duration between treatment and onset of skin lesions was 23 months (IQR, 15–41 months). The majority of patients (9/16) reported that the skin lesions occurred within 24 months after VL treatment.

Hypo-pigmented macules/plaques were the most common hypo pigmented lesions and were present first on the face (11/16), and upper and lower limbs were also affected. In most (13/16) cases reported, skin lesions appeared gradually from face to lower extremities and was associated with itching on erythema when exposed in sun light. Only 4 patients with skin lesions had sought medical treatment mainly for cosmetic purposes. We didn't find any PKDL cases with hepato-and/or splenomegaly and other associated complications such as post-kala-azar conjunctivitis or uveitis.

### Risk and prevalence of PKDL

**Figure 2:**
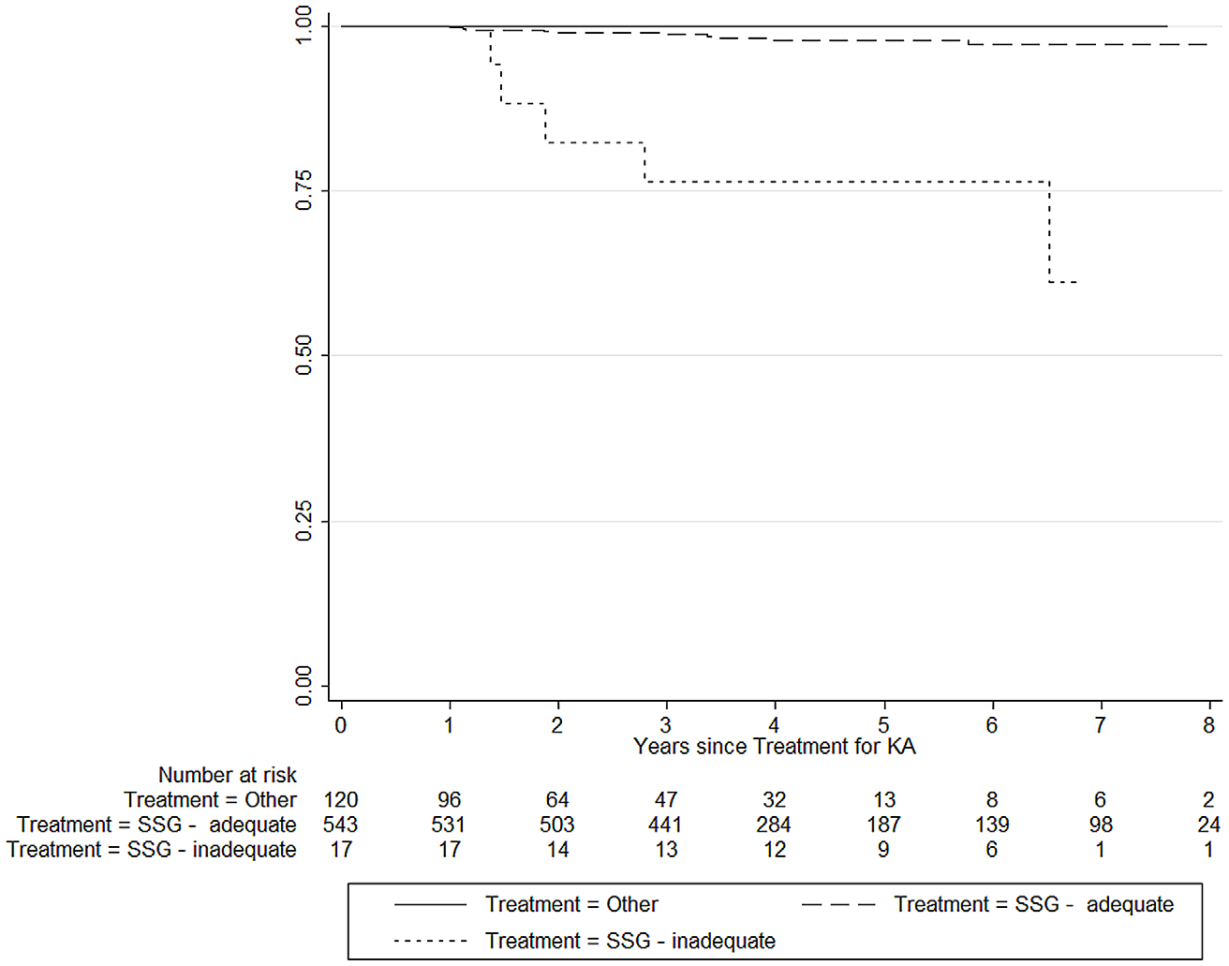
Risk of PKDL in three VL-treatment groups, Kaplan-Meier survival analysis. Kaplan-Meier survival analysis for three types of VL treatment.

Overall, the risk to develop PKDL was 1.4% within two years after VL treatment, 2.5% within 4 years and 3.6% within 8 years (see Table 1). The risk of PKDL by treatment (adequate SSG, inadequate SSG and other treatments is shown in Figure 2. In the SSG treated group alone (560 patients) the prevalence rate of PKDL was 2.9%. In the univariate analysis, PKDL was significantly associated with unsupervised treatment at government/private hospitals (OR = 16.28; 95% CI 2.48–689.00) with inadequate SSG treatment in the past (OR = 19.77; 95% CI 4.66–75.00). Both findings remained independently significant in the multiple logistic regression model. In the univariate analysis, the risk of PKDL appeared higher in private hospitals (OR = 13.4; 95% CI 0.7–802.7), but this finding was not significant and was not supported when corrected for treatment. None of the other assessed risk factors (age, sex, hospitalization during treatment with SSG) were significantly associated with PKDL (Table 2).

**Table 2:** Risk factors for PKDL development in past treated VL patients, exact logistic regression.

Factors	Past VL (no.)	PKDL	Unadjusted ORα	95% CIβ	Adjusted OR	95% CI
		no. (%)				
Age (year) median (IQR)δ	680	28 (15;40)				
≤14 year	167	3 (1.8)	referent			
15–29 year	194	6 (3.1)	1.74	0.37–10.93		
30–44 year	189	4 (2.1)	1.18	0.20–8.18		
≥45 year	130	3 (2.3)	1.29	0.17–9.80		
Gender						
Male	370	8 (2.2)	referent			
Female	310	8 (2.6)	1.20	0.39–3.71		
Drug used for VL treatment						
Adequate SSGσ treatment	543	11 (2.0)	referent		referent	
Inadequate SSG treatment (≤20 injection)	17	5 (29.4)	19.77	4.66–75.00	11.68	2.71–45.47
Other treatment (Miltefosine & Amphotericin B)	120	0 (0.0)	0.29*	0.00–1.80	0.67*	0.00–4.59
Supervised treatment with SSG at BPKIHS						
Adequate treatment	247	1 (0.4)				
Inadequate treatment (≤20 injection)	0	0 (0.0)				
Unsupervised treatment with SSG at other places (Govt. & pvt)						
Adequate treatment	296	10 (3.4)				
Inadequate treatment (≤20 injection)	17	5 (29.4)				
Hospitalization during treatment with SSG						
No	334	12 (3.6)	referent			
Yes	226	4 (1.8)	0.45	0.14–1.40		

**α:** OR : odds ratio;

**β:** CI: confidence interval;

**δ:** IQR: Inter quartile range;

**σ:** SSG : Sodium stibogluconate.

*: Median unbiased estimates (MUE).

## Discussion

There have been few reports and studies on PKDL from Nepal [7], [16]–[18], and the frequency and risk factors of PKDL have not been studied previously. When re-examining a group of patients who were treated for VL in the previous ten years, we have found 2.4% having PKDL, and 2.9% in the sub-group of those treated with SSG in particular. The risk estimate for PKDL after VL was 1.4% within 2 years, and 3.6% within 8 years based on Kaplan–Meier analysis. This risk is lower than that reported in other VL-endemic areas in the Indian subcontinent [3], [8]–[9], [17]. Still, risk estimates reported are hard to compare due to the unequal follow up times. The median time from VL treatment to PKDL onset was 23 months (IQR, 15–41 months). PKDL was more common in those with incomplete VL treatment and in settings with little treatment supervision.

A limitation of our study is that enrolment in the cohort was based on data obtained from one tertiary hospital and governmental health facilities. It is generally assumed that VL is underreported [19], as patients may be seeking treatment in the private sector. However, in our region in Nepal only few VL patients attend private clinics for VL treatment as anti-VL drugs are provided free-of-charge in the public health structures and are not available in private pharmacies. No cases of former VL treatment through private practitioners or pharmacies have ever been reported to the staff in the VL treatment centre at BPKIHS (personal communication). This could possibly help explaining why the frequency of PKDL in Nepal is lower than in the other reports from the Indian subcontinent.

Secondly, we assessed the presence of PKDL in 2010 in a cohort of patients diagnosed with VL between 2000 up to 2009; time of follow-up is variable, which makes the analysis more complex. The national VL control program initiated miltefosine-based treatment protocols in 2007, and the 54 pVL cases treated with miltefosine have been regrouped for the purpose of this analysis with those who received Amphotericin. Though none of the VL patients in this group (i.e. Miltefosine or Amphotericin B) developed PKDL, follow-up time for miltefosine was definitely shorter than for the other drugs and therefore no final conclusions should be drawn yet about this drug [8]. Cases of PKDL in patients treated with Miltefosine have already been reported from India [20].

In another PKDL study conducted in the Fulbaria sub-district of Mymensingh district in Bangladesh, a cross-sectional survey was used to detect all past or active VL cases and active PKDL cases in a time period (2002–2007) and calculated incidences [8]. In this study clinical signs of PKDL were found in 9.8% of the 813 identified pVLs, almost 2 times higher than the proportion of suspects found in our study (5.4%) while the time period studied was shorter. The authors mention however that parasitological confirmation of PKDL was only done in 10 suspected PKDL cases and confirmation was only obtained in 4 of these, a confirmation rate that was similar to ours. In another study in Trishal subdistrict of Mymensingh in Bangladesh without restriction in time of onset of VL [9] 52 PKDL suspects were identified for 235 pVLs, of which 18 (7.6%) were as probable cases and 9 (3.8%) were ultimately confirmed. Neither of both studies estimated the risk for PKDL over time using survival analysis.

The single cross-sectional dermatological assessment may have made us underestimate the true incidence of PKDL, though no self-healing has been described for this pathology. No pVL patient in the study reported any symptoms of PKDL that since had disappeared - spontaneously or after treatment. One case of PKDL without antecedents of VL treatment was identified during the survey and confirmed at BPKIHS. In the cross-sectional surveys in Bangladesh, PKDL without previous VL accounted for 10% of all PKDL cases [8]–[9]. In Nepal an earlier study at BPKIHS reviewing the 22 cases of PKDL that were diagnosed between 1998 and 2000, only 1 case had no clinical VL history [7]. It is thus unlikely that a high number of PKDL cases without previous VL have been missed in our survey.

Median time of onset and clinical features of PKDL patients were all consistent with the findings from India and Bangladesh [7], [12], [21]. All 16 PKDL cases had lesions in the face, but only four had sought treatment for PKDL. All four were female and 3 were unmarried.

In our study, the risk analysis only included data on VL history and treatment, and did not look into clinical markers such as HIV- and nutritional status, or immunological markers such as cytokines [22]–[23]. HIV prevalence is low in Nepal and even more so in the rural population affected by VL. Inadequate treatment received by pVLs in the past represented the most significant risk factor for PKDL (OR 11.68, 95% CI 2.71–45.47). This is in line with earlier findings from Sudan where inadequate dosage and duration, and irregular treatment were important predictors for PKDL [6], [23]. Without supervision of treatment and adherence counselling, patients may indeed abandon treatment earlier than prescribed, as clinical improvement usually appears within the first week of VL treatment, and there is little incentive to continue the painful intramuscular injections with SSG. In Nepal, VL treatment is provided for free to overcome financial barriers, but in some cases, treatment interruption was reportedly due to stock shortages of SSG at the hospital level. Treatment compliance should therefore be correctly monitored by clinicians and programmes, and all patients should be counselled about the importance of treatment adherence. This is important not only to avoid development of PKDL but also to reduce risks of treatment failure and development of drug resistance.

It should be clear that PKDL is a multi-factorial phenomenon of complex origin [5] whereby drug related factors are not the only reasons for PKDL development. Host and parasite factors need to be further elucidated [24].

In conclusion, the occurrence of PKDL after VL treatment is relatively rare in Nepal compared to the two neighbouring countries involved in the VL Elimination Initiative. SSG, ambulatory treatment at government health facilities and inadequate treatment for VL in the past were significantly associated with PKDL. Counselling and supervision of treatment adherence in VL seems therefore essential to reduce PKDL incidence in the future, even if SSG is no longer used in Nepal. Reporting of cases of PKDL should be an integral part of the surveillance and monitoring system. Early identification can be improved by counselling VL patients on the risks and the signs of PKDL during their treatment

Ultimately, the burden of PKDL can only be efficiently tackled if a more effective, affordable and short treatment can be offered to the patients.
